# Molecular characterization of a clinical ST145 *Klebsiella oxytoca* strain co-producing KPC-2 and IMP-96 carbapenemases

**DOI:** 10.1128/msystems.01529-25

**Published:** 2025-12-17

**Authors:** Hao Liu, Chao Yan, Sibo Wang, Juntian Jiang, Meiling Jiao, Fupin Hu, Xuesong Xu

**Affiliations:** 1 Department of Laboratory Medicine, China-Japan Union Hospital of Jilin University 74569, Changchun, China; 2 Institute of Antibiotics, Huashan Hospital, Fudan University 198171, Shanghai, China; 3 Joint Laboratory of Hospital & Enterprise for Pathogen Diagnosis of Drug-resistant Bacterial Infections and Innovative Drug R&D, Shanghai, China; 4 Key Laboratory of Clinical Pharmacology of Antibiotics, Ministry of Health 659796 , Shanghai, China; University of Maryland School of Medicine, Baltimore, Maryland, USA

**Keywords:** *Klebsiella oxytoca*, carbapenemase, *bla*_KPC-2_, *bla*_IMP-96_

## Abstract

The objective of this study was to investigate the antimicrobial resistance phenotype and genetic characteristics of a clinical ST145 *Klebsiella oxytoca* isolate co-producing KPC-2 and IMP-96 carbapenemases. The isolate was first identified by MALDI-TOF MS. PCR and Sanger sequencing were used to detect carbapenem resistance genes. Antimicrobial susceptibility testing was performed using broth microdilution. Whole genome sequencing was carried out using Illumina and Nanopore platforms. Conjugation experiments and comparative genomic analysis were used to assess plasmid transferability and the genetic context of resistance genes. A total of 103 *K*. *oxytoca* genome sequences were retrieved from public databases and, together with the isolate from this study, used to construct a core genome single nucleotide polymorphism (SNP)-based phylogenetic tree. The antimicrobial resistance genes carried by each strain were also analyzed. Antimicrobial susceptibility testing revealed that *K. oxytoca* K31 was resistant to cephalosporins, carbapenems, and ceftazidime-avibactam (MIC range: 8 to >64 µg/mL), but susceptible to amikacin, meropenem-vaborbactam, aztreonam-avibactam, eravacycline, tigecycline, and colistin. Whole genome sequencing analysis showed that the strain was classified as ST 145. The *bla*_KPC-2_ and *bla*_IMP-96_ genes were located on IncFIB(K)-like and IncM1 plasmids, respectively. Conjugation experiments confirmed that both plasmids carrying carbapenem resistance genes were transferable to recipient strain *Escherichia coli* J53 and conferred carbapenem resistance. Comparative genomic analysis indicated that the *bla*_KPC-2_ gene was located in the variable region of a Tn*3* family transposon, whereas the *bla*_IMP-96_ gene was embedded in a gene cassette captured by the *IntI1*. Genomic analysis of 104 ST145 *K. oxytoca* isolates revealed that 94.2% (98/104) harbored at least one carbapenem resistance gene. This study is the first report of a clinical isolate of ST145 *K. oxytoca* co-producing *bla*_KPC-2_ and *bla*_IMP-96_. Both resistance genes are located on mobile genetic elements that can be transferred between different bacterial species, facilitating the spread of antimicrobial resistance.

**IMPORTANCE:**

Carbapenem-resistant *Klebsiella oxytoca* has been increasingly reported worldwide; however, isolates co-producing both class A and class B carbapenemases remain rare. This study reported a clinical ST145 *K. oxytoca* isolate co-harboring the *bla*_KPC-2_ and *bla*_IMP-96_ resistance genes, which exhibited high-level resistance to both carbapenems and ceftazidime-avibactam. The two carbapenemase genes were located on conjugative plasmids separately with autonomous transfer capability. Genetic context analysis revealed that both resistance genes were embedded in mobile genetic elements, which likely mediate their capture and horizontal transfer across bacterial species. The widespread distribution of such mobile elements carrying resistance genes accelerates the evolution of multidrug-resistant bacteria. Genomic analysis of global ST145 *K. oxytoca* strains further indicated that this sequence type represents a high-risk, multidrug-resistant clonal lineage with significant public health implications. Enhanced surveillance and screening of ST145 *K. oxytoca* are warranted to limit its further global spread.

## INTRODUCTION

*Klebsiella oxytoca* is a non-motile, encapsulated, Gram-negative bacterium that can colonize the gastrointestinal tract of healthy individuals (1) and is also found in other human body sites, such as the skin and oropharynx (2). The pathogenicity of *K. oxytoca* is closely related to the immune status of the host and is currently considered an opportunistic pathogen associated with nosocomial infections (3, 4). *K. oxytoca* can cause a range of infections, including antibiotic-associated hemorrhagic colitis, bloodstream infections, urinary tract infections, pneumonia, and intra-abdominal infections (5–9). According to the 2024 data from the China Antimicrobial Surveillance Network (CHINET) (https://www.chinets.com/), the overall detection rate of *K. oxytoca* among the 458,271 clinical isolates is approximately 1%. In recent years, with the widespread use of carbapenems, carbapenem-resistant *K. oxytoca* strains have been increasingly reported (10, 11), posing a new threat to public health.

Carbapenem resistance in *K. oxytoca* is primarily mediated by the production of carbapenemases. According to the Ambler classification system, the most common class A carbapenemase is *Klebsiella pneumoniae* carbapenemase (KPC), while class B enzymes include Verona integron-encoded metallo-β-lactamase (VIM) and imipenemase (IMP), and class D enzymes are OXA-48-like (2). KPC, the most common class A serine carbapenemase, can hydrolyze carbapenems, penicillins, cephalosporins, and aztreonam through a serine-based active site (12). However, its activity can be inhibited by novel β-lactamase inhibitors, such as avibactam (13). To date, Gram-negative bacteria harboring the *bla*_KPC_ gene are widely disseminated throughout the world (14). Among the class B metallo-β-lactamases, the IMP-type carbapenemase is encoded by the *bla*_IMP_ gene, and its enzymatic activity is mediated by zinc ions to hydrolyze the β-lactam ring. Unlike KPC, these enzymes can be inhibited by metal chelators such as EDTA (15). Molecular studies have shown that *bla*_IMP_ is frequently transferred horizontally between different Gram-negative bacterial species via class 1 and class 3 integrons (16). Infections caused by carbapenem-resistant bacteria are associated with higher mortality rates and represent a major challenge for clinical management.

Strains producing multiple carbapenemases pose significant challenges in clinical treatment, particularly those co-producing class B metallo-β-lactamases and KPC, as they render most β-lactam antibiotics and β-lactam/β-lactamase inhibitor combinations ineffective (17, 18). In 2011, Chinese researchers first reported a clinical case of *K. oxytoca* co-producing KPC-2 and IMP-8 carbapenemases (19). Notably, the novel *bla*_IMP_ mutant, *bla*_IMP-96_, was first identified in the chromosomal genome of *Stenotrophomonas* spp. (20). In this study, we describe a clinical isolate of *K. oxytoca* co-producing KPC-2 and IMP-96 carbapenemases. Molecular epidemiological tracking suggested that the *bla*_IMP-96_ gene might have been horizontally transferred across species, possibly originating from *Stenotrophomonas* spp. and disseminated to *Enterobacter hormaechei* and *K. oxytoca* through class 1 integron-based mobile genetic elements. Using whole genome sequencing, conjugation experiments, and antimicrobial susceptibility profiling, we have elucidated the genetic context of these resistance genes and their potential for dissemination. This finding not only reveals the cross-species transmission pathway of a novel carbapenemase gene but also provides crucial molecular epidemiological evidence for the control and prevention of hospital-acquired infections caused by multidrug-resistant pathogens.

## MATERIALS AND METHODS

### Strains and case information

The *K. oxytoca* K31 strain was isolated from the puncture drainage fluid of an epidural abscess in a 72-year-old male patient. This patient was admitted to the emergency department of the China-Japan Union Hospital of Jilin University in 2020 for acute onset of cerebral hemorrhage, with concomitant bilateral pneumonia. The patient underwent craniectomy with decompression, hematoma evacuation, and ventriculostomy for drainage. Prophylactic antimicrobial treatment was started with piperacillin-sulbactam (5.0 g Q12h). On postoperative day 2, the patient underwent a tracheotomy due to worsening pneumonia and sputum retention. By day 7, the patient developed fever, and cerebrospinal fluid analysis revealed a significantly elevated white blood cell count, consistent with secondary intracranial infection. Vancomycin (1.0 g Q12h) and meropenem (1.0 g Q6h) were added to control the infection. However, on postoperative day 11, repeat lumbar puncture indicated unresolved infection, with a maximum body temperature of 39.2°C and the development of an epidural abscess around the cranial incision. *K. oxytoca* K31 was isolated from the abscess drainage. Antimicrobial susceptibility testing showed that the strain was resistant to imipenem, meropenem, and ceftazidime-avibactam. The patient eventually discharged himself due to critical illness. The bacterial species was initially identified using matrix-assisted laser desorption/ionization time-of-flight mass spectrometry (MALDI-TOF MS) (BioMérieux, France), and confirmed by whole genome sequencing.

### Antimicrobial susceptibility testing and carbapenemase detection

According to the guidelines of the Clinical and Laboratory Standards Institute (CLSI) (21), the minimum inhibitory concentrations (MICs) of antimicrobial agents were determined using the broth microdilution method. The tested antimicrobial agents included imipenem, meropenem, meropenem-vaborbactam, aztreonam-avibactam, ceftazidime-avibactam, piperacillin-tazobactam, ceftazidime, ceftriaxone, cefepime, aztreonam, amikacin, ciprofloxacin, levofloxacin, colistin, tigecycline, and eravacycline. Quality control procedures and interpretation of results were performed according to the CLSI 2024 breakpoint criteria (21). Antimicrobial susceptibility results for colistin and aztreonam-avibactam were interpreted according to the guidelines of European Committee on Antimicrobial Susceptibility Testing (EUCAST) (22). The MICs of tigecycline and eravacycline were interpreted according to the *Enterobacterales* breakpoints defined by the U.S. Food and Drug Administration (FDA) (23). *Escherichia coli* ATCC 25922 and *Pseudomonas aeruginosa* ATCC 27853 were used as quality control strains. Carbapenemase phenotypic detection was performed using meropenem (10 µg) susceptibility disks alone, and in combination with phenylboronic acid (PBA), EDTA, or both PBA and EDTA, as previously described (24).

### Whole genome sequencing and data analysis

Genomic DNA was extracted from the bacterial strain using a DNA extraction kit according to the manufacturer’s instructions. Whole genome sequencing was performed using both short-read sequencing technology (Illumina, San Diego, CA, USA) and long-read sequencing technology (Nanopore, Oxford, UK). Raw sequencing data were filtered for quality and trimmed for read length using Sickle (GitHub). Hybrid assembly was performed with SPAdes version 3.12.0 to generate a high-quality draft genome. Multi-locus sequence typing (MLST) typing was performed using the BIGSdb-Pasteur MLST database (https://bigsdb.pasteur.fr/), while plasmid replicon types and antimicrobial resistance genes were identified using the PlasmidFinder, Pmlst, and ResFinder databases available at the Center for Genomic Epidemiology (http://www.genomicepidemiology.org/services/). Capsule serotypes and virulence genes were analyzed using Kleborate (http://github.com/katholt/Kleborate) (25) and VFDB (https://www.mgc.ac.cn/VFs/) (26). Open reading frames (ORFs) were predicted and annotated using RAST version 2.0 and BLAST (https://blast.ncbi.nlm.nih.gov/Blast.cgi).

### Conjugation experiment and plasmid sequencing

*Escherichia coli* J53, a strain resistant to sodium azide, was used as the recipient strain to evaluate plasmid transferability using a standard liquid conjugation method. During the experiment, donor and recipient strains were mixed at a 1:1 cell ratio and co-cultured in pre-warmed lysogeny broth (LB), Luria-Bertani formula, at 37°C, followed by co-incubation at 37°C for 18 h. Transconjugants were selected on Mueller–Hinton (MH) agar plates containing dual selective agents (aztreonam 8 mg/L + sodium azide 100 mg/L, or ceftazidime-avibactam 4 mg/L + sodium azide 100 mg/L). Positive colonies growing on the selective media were rapidly identified using MALDI-TOF MS. PCR and PCR-based Sanger sequencing were used to confirm the presence of *bla*_KPC-2_ and *bla*_IMP-96_ genes in the transconjugants. Plasmid DNA from the transconjugants was extracted using the Qiagen Midi Kit (Qiagen, Hilden, Germany) and further verified by short-read sequencing on the Illumina platform (San Diego, CA, USA).

### Phylogenetic analysis

To further investigate the evolutionary relationships among ST145 *K. oxytoca* isolates, 103 publicly available whole genome sequences were downloaded from the Bacterial and Viral Bioinformatics Resource Center (BV-BRC). These genomes, together with the isolate characterized in this study, were used to construct a single nucleotide polymorphism (SNP)-based phylogenetic tree. SNP distances between bacterial genomes were calculated using Snippy v4.6.0 (https://github.com/tseemann/snippy), run with default parameters. The phylogenetic tree was constructed using FastTree and subsequently visualized and refined via the ChiPlot platform (27). Antimicrobial resistance genes were screened using ABRicate v1.0.1(https://github.com/tseemann/abricate) against the ResFinder database (≥80% identity, ≥90% coverage).

## RESULTS

### Characteristics of *K. oxytoca* K31 clinical strain

Antimicrobial susceptibility testing revealed that *K. oxytoca* K31 was highly resistant to carbapenems, β-lactams, β-lactam/β-lactamase inhibitor combinations, and quinolones. Specifically, the strain was resistant to imipenem (MIC = 16 µg/mL), meropenem (MIC = 8 µg/mL), aztreonam (MIC >128 µg/mL), ceftazidime (MIC >32 µg/mL), ceftriaxone (MIC >32 µg/mL), cefepime (MIC = 32 µg/mL), piperacillin-tazobactam (MIC >256 µg/mL), ceftazidime-avibactam (MIC >64 µg/mL), ciprofloxacin (MIC >8 µg/mL), and sitafloxacin (MIC = 2 µg/mL). However, amikacin (MIC ≤1 µg/mL), meropenem-vaborbactam (MIC = 4 µg/mL), aztreonam-avibactam (MIC = 0.25 µg/mL), eravacycline (MIC = 0.25 µg/mL), tigecycline (MIC = 0.25 µg/mL), and colistin (MIC = 0.25 µg/mL) exhibited good *in vitro* activity against K31 (Table 1).

**TABLE 1:** Antimicrobial susceptibility of *K. oxytoca* K31, transconjugants, and recipient strain

Antibiotic	MIC (μg/mL)					
	*K. oxytoca* K31 (KPC-2 + IMP-96)	*E. coli* J53 (KPC-2)	*E. coli* J53 (IMP-96)	*E. coli* J53	*E. coli* ATCC 25922	*P. aeruginosa* ATCC 27853
Imipenem	16	2	0.5	0.125	≤0.06	1
Meropenem	8	4	4	≤0.03	≤0.03	0.125
Meropenem-vaborbactam	4	≤0.03	4	≤0.03	≤0.03	0.06
Aztreonam-avibactam	0.25	0.25	0.125	0.06	≤0.03	2
Ceftazidime-avibactam	>64	0.5	>64	0.25	0.06	1
Piperacillin-tazobactam	>256	128	64	4	≤2	≤2
Ceftazidime	>32	16	>32	0.5	≤0.25	1
Ceftriaxone	>32	32	>32	≤0.25	≤0.25	8
Cefepime	32	8	32	0.5	≤0.25	0.5
Aztreonam	>128	128	≤1	≤1	≤1	2
Amikacin	≤1	2	2	2	≤1	2
Ciprofloxacin	>8	≤0.06	≤0.06	≤0.06	≤0.06	0.25
Sitafloxacin	2	≤0.06	≤0.06	≤0.06	≤0.06	0.125
Colistin	0.25	0.5	0.25	0.125	0.25	1
Tigecycline	0.25	0.125	0.125	0.125	≤0.06	4
Eravacycline	0.25	0.125	0.125	0.06	≤0.06	2

### Detection of carbapenemase genes and conjugation experiment

PCR-based sequencing results revealed that *K. oxytoca* K31 simultaneously carries two carbapenemase-encoding genes, *bla*_KPC-2_ and *bla*_IMP-96_. Conjugation experiments confirmed that plasmids carrying carbapenem resistance genes were successfully transferred to *E. coli* J53. Transconjugants grown on selective medium containing aztreonam (8 mg/L) and sodium azide (100 mg/L) were verified by PCR to be *bla*_KPC-2_-positive, whereas colonies growing on medium containing ceftazidime-avibactam (4 mg/L) and sodium azide (100 mg/L) were confirmed to be *bla*_IMP-96_-positive. The conjugation frequency of the *bla*_KPC-2_-positive plasmid was 3.6 × 10⁻⁶, while that of the *bla*_IMP-96_-positive plasmid was 5.6 × 10⁻⁵ (calculated according to the number of conjugants per initial donor bacteria). Antimicrobial susceptibility testing showed that KPC-positive transconjugants were resistant to meropenem (MIC = 4 µg/mL), intermediate to imipenem (MIC = 2 µg/mL), and sensitive to ceftazidime-avibactam (MIC = 0.5 µg/mL). IMP-positive transconjugants were resistant to meropenem (MIC = 4 µg/mL) and ceftazidime-avibactam (MIC >64 µg/mL) but sensitive to imipenem (MIC = 0.5 µg/mL). Compared with the recipient strain *E. coli* J53, the MICs of imipenem, meropenem, and ceftazidime-avibactam in KPC-2-positive transconjugants increased by at least 16, 128, and 2-fold, respectively. For *bla*_IMP-96_-positive transconjugants, the MICs of imipenem, meropenem, and ceftazidime-avibactam increased by at least 4, 128, and 256-fold, respectively (Table 1).

### Whole genome sequencing analysis of plasmid sequences harboring *bla*_KPC-2_ and *bla*_IMP-96_ and their genetic context

**Fig 1:**
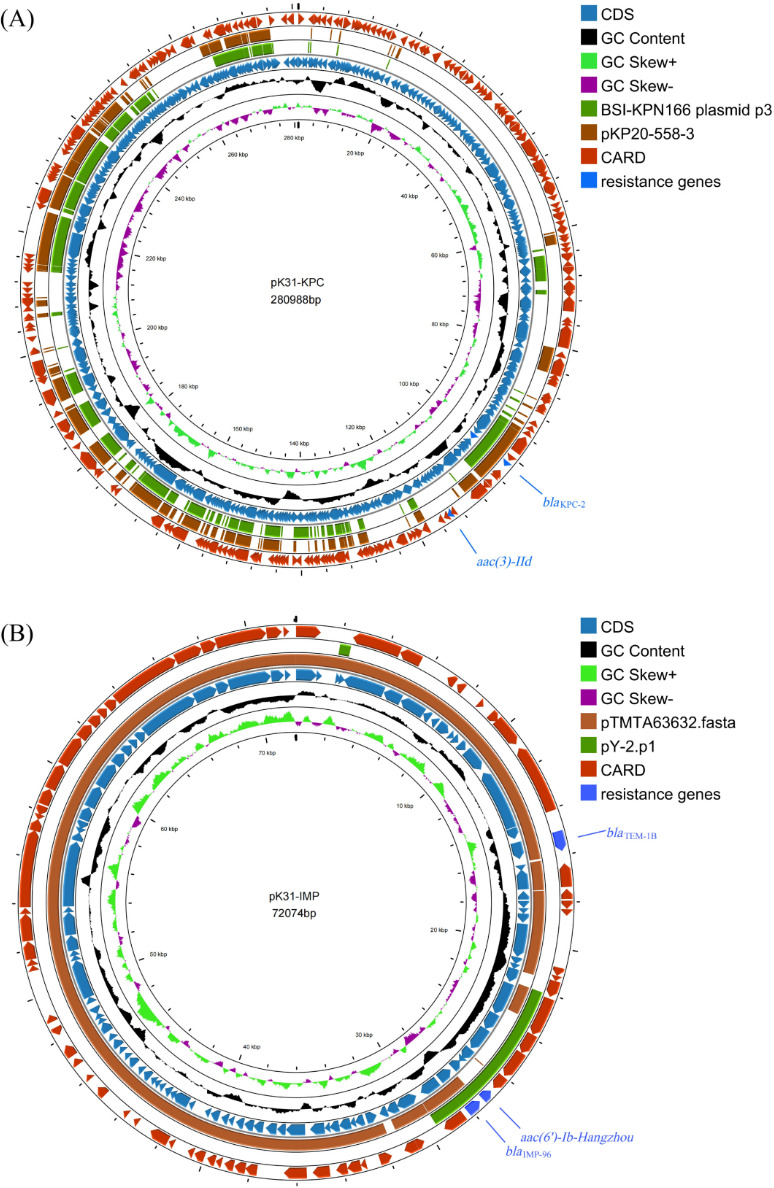
(**A**) Circular comparison of plasmid pK31-KPC with plasmids BSI-KPN166 plasmid p3 (CP102106) and pKP20-558-3 (CP096266). (**B**) Circular comparison of plasmid pK31-IMP with plasmids pY-2.p1 (CP090797) and pTMTA63632 (AP019667). The different colors in the rings represent different plasmids. Resistance genes are labeled in blue.

**Fig 2:**
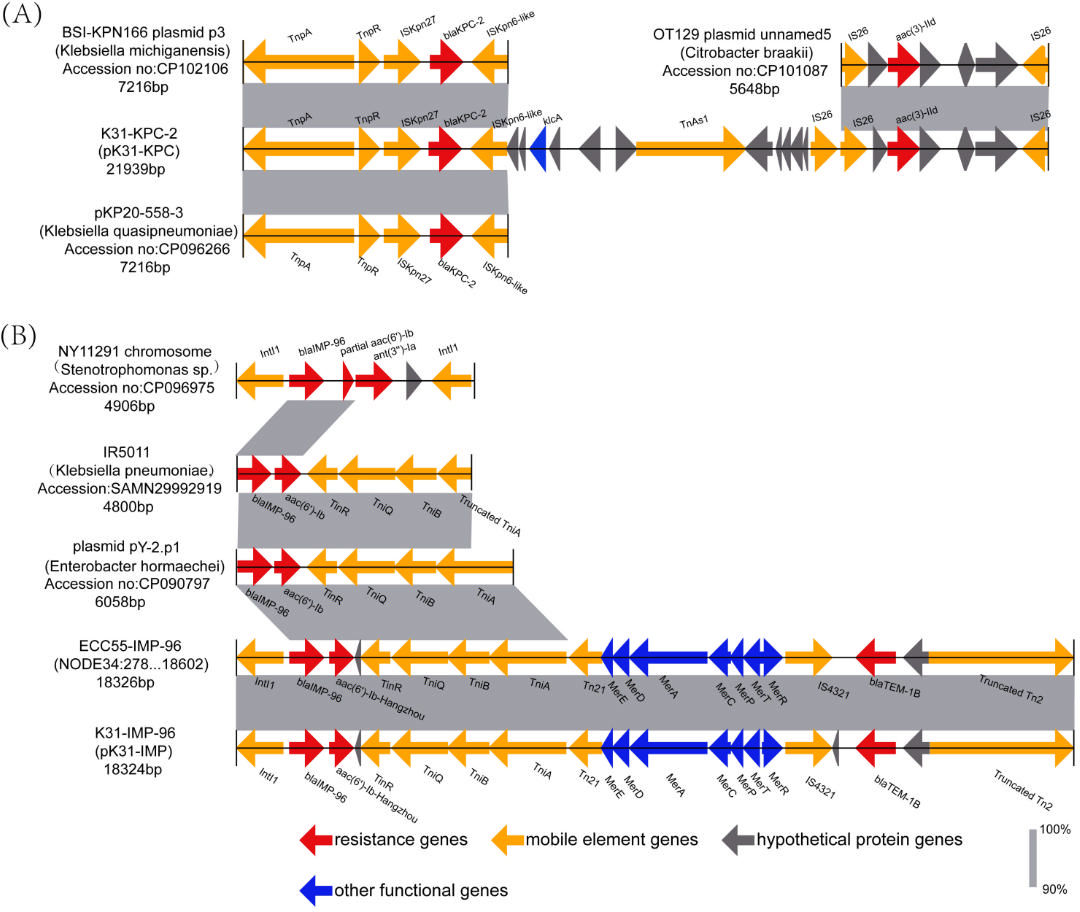
Genetic context of plasmid-borne resistance genes.(**A**) Comparison of the genetic environment of *bla*_KPC-2_ on plasmid pK31-KPC with that of plasmids BSI-KPN166 plasmid p3 (CP102106) and pKP20-558-3 (CP096266). The genetic environment of the *Aac (3)-IId* gene on plasmid pK31-KPC is compared with that of plasmid OT129 plasmid unnamed5 (CP101087). (**B**) Comparison of the genetic environment of *bla*_IMP-96_ on plasmid pK31-IMP with that of the chromosomal region of NY11291 (CP096975), IR5011(SAMN29992919), plasmid pY-2.p1 (CP090797), and ECC55-IMP-96 (NODE34). Genes are represented by arrows and are color-coded based on their functional categories.

Whole genome sequencing analysis revealed that the *K. oxytoca* K31 strain (accession number: GCA_051123605.1) belongs to ST145, with a predicted capsular serotype of KL128. The total genome size is 6,466,832 bp, including a 6,023,973 bp chromosome and eight circular plasmids of sizes 280,988, 72,074, 58,767, 8,613, 7,066, 5,890, 5,413, and 4,048 bp. Several common antimicrobial resistance genes were identified, including the carbapenemase genes *bla*_KPC-2_ and *bla*_IMP-96_, the β-lactam resistance genes *bla*_TEM-1B_ and *bla*_OXY-2-9_, the aminoglycoside resistance genes *aac (3)-IId* and *aac(6')-Ib-Hangzhou*, and the quinolone resistance gene *qnrS1*. Furthermore, several virulence factors were detected, including *fyuA, ybtE, ybtT, ybtU, irp1, irp2, ybtA, ybtP, ybtQ, ybtX, ybtS, ompA, entA, entB,* and *yagZ/ecpA* (Table 2). Sequencing analysis identified the conjugative plasmid pK31-KPC, which is 280,988 bp in size and contains three potential origins of replication. On comparison with the PlasmidFinder database, we found that pK31-KPC had the highest similarity to IncFIB(K) plasmids (98.75% identity). Therefore, it was classified as an IncFIB(K)-like plasmid. pK31-KPC contains 346 ORFs, with the *bla*_KPC-2_ and *aac (3)-IId* genes responsible for carbapenem and aminoglycoside resistance, respectively (Fig. 1A). Blast comparison results showed that the multidrug-resistant region containing *bla*_KPC-2_ and *aac (3)-IId* was not highly homologous to any known plasmids in existing databases. The genetic environment surrounding *bla*_KPC-2_ showed 99% identity and 99% query coverage with the plasmid p3 from *Klebsiella michiganensis* (accession number: CP102106) and plasmid pKP20-558-3 from *Klebsiella quasipneumoniae* (accession number: CP096266). The surrounding genetic structure was *tnpA-tnpR*-IS*Kpn27-bla*_KPC-2_-IS*Kpn6-like*. The genetic environment of *aac (3)-IId* showed high similarity (99% identity and query coverage) to the plasmid OT129 unnamed5 from *Citrobacter braakii* (accession number: CP101087) (Fig. 2).

**TABLE 2:** Chromosomal and plasmid characteristics of *K. oxytoca* K31

Chromosome/ plasmid	Size (bp)	GC content (%)	Plasmid incompatibility group	Antibiotic resistance genes	Virulence factor genes
Chromosome	6,023,973	55%		*bla*_OXY-2-9_	*fyuA, ybtE, ybtT, ybtU, irp1, irp2, ybtA, ybtP, ybtQ, ybtX, ybtS, ompA, entA, entB, yagZ/ecpA*
pK31-KPC	280,988	53%	IncFIB(K)-like	*bla*_KPC-2_, *aac (3)-IId*	
pK31-IMP	72,074	53%	IncM1	*bla*_IMP-96_, *bla*_TEM-1B_, *aac(6')-Ib-Hangzhou*	
pK31-3	58,767	52%	repB(R1701)	*qnrS1*	
pK31-4	8,613	53%	–^*a*^		
pK31-5	7,066	51%	–		
pK31-6	5,890	46%	–		
pK31-7	5,413	51%	–		
pK31-8	4,048	30%	–		

^*a*^ –, no corresponding plasmid type could be matched in the currently available plasmid databases.

Plasmid pK31-IMP, an IncM1-type plasmid of 72,074 bp, contains 98 ORFs. This plasmid harbors the *bla*_IMP-96_, *bla*_TEM-1B_, and *aac(6')-Ib-Hangzhou* resistance genes (Fig. 1B), which confer resistance to carbapenems, β-lactams, and aminoglycosides, respectively. Blast comparison analysis showed that plasmid pK31-IMP shares 99% identity and 93% query coverage with plasmid pTMTA63632 from *Klebsiella pneumoniae* strain TA6363 (accession number: AP019667). Comparative genetic structure analysis revealed that the *bla*_IMP-96_ gene is located on the chromosome of *Stenotrophomonas* spp. strain NY11291 (accession number: CP096975) isolated in Beijing, China, flanked on both sides by integron *IntI1*. In *Klebsiella pneumoniae* strain IR5011 (accession number: SAMN29992919), also isolated in Beijing in the same year, *bla*_IMP-96_ was identified with the right-side *intI1* replaced by a transposon-associated gene cluster (*TniR–TniQ–TniB–*truncated *TniA*). A similar structural arrangement was subsequently observed on plasmid pY-2.p1 (accession number: CP090797) of *Enterobacter hormaechei* Y-2 isolated in Hohhot, China. Notably, we also found that *Enterobacter hormaechei* strain ECC55, which also carries *bla*_IMP-96_, shares an identical multidrug-resistant genetic region with K31. This region has the genetic structure: *IntI1-bla*_IMP-96_*-aac(6')-Ib-Hangzhou-TniR-TniQ-TniB-TniA-Tn21-MerE-MerD-MerA-MerC-MerP-MerT-MerR-*IS*4321-bla*_TEM-1B_*-*Truncated Tn*2* (Fig. 2).

### Phylogenetic tree and resistance genes of ST145 *K. oxytoca*

**Fig 3:**
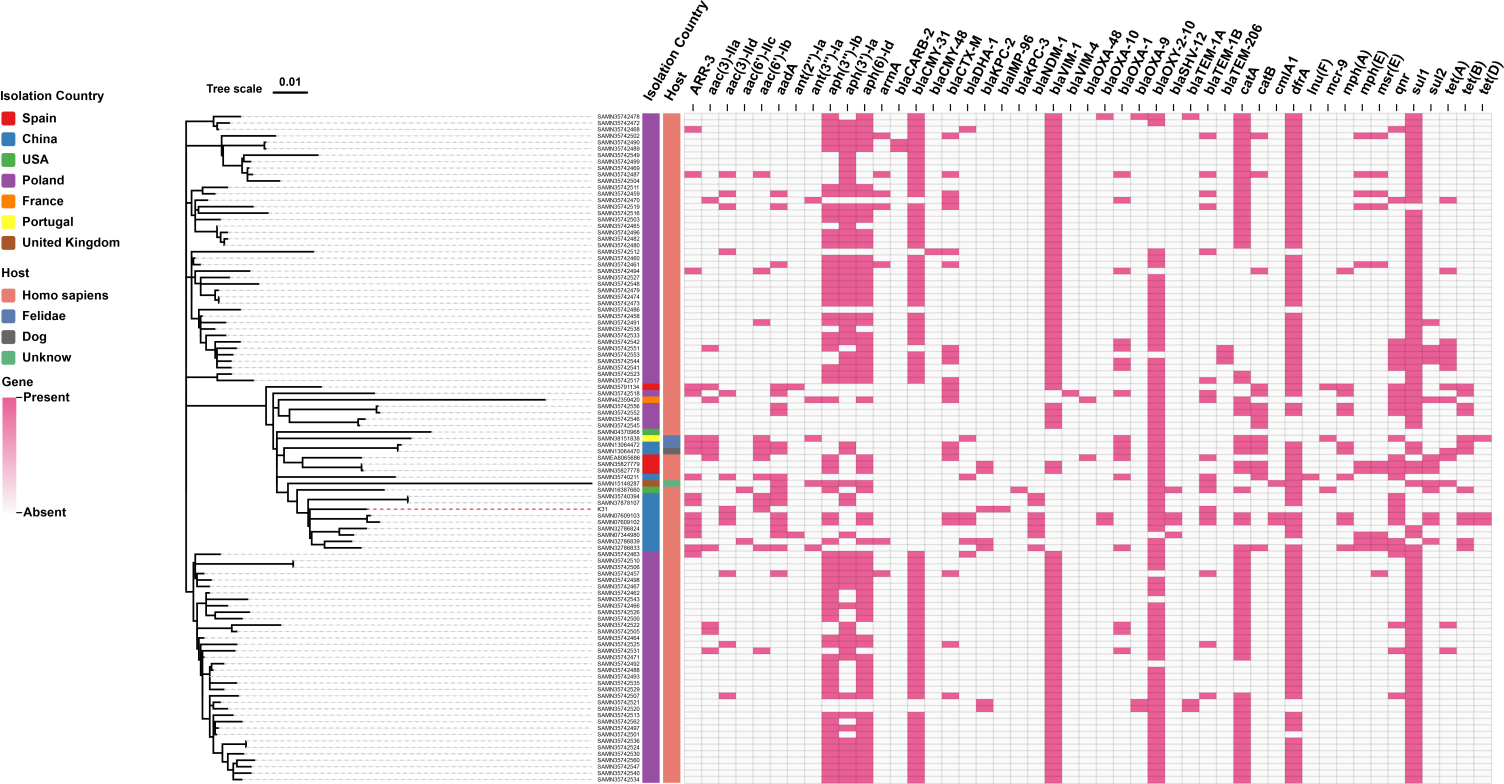
Phylogenetic tree of 104 ST145 *K. oxytoca* isolates and the distribution of their associated antimicrobial resistance genes.

A total of 103 whole-genome sequences of ST145 *K. oxytoca* isolates were retrieved from public databases and, together with the clinical isolate K31 from this study, used to construct a core genome SNP-based phylogenetic tree and to analyze the antimicrobial resistance gene profiles. The 104 isolates included in the analysis originated from seven countries: Poland (*n* = 83), China (*n* = 12), Spain (*n* = 4), the United States (*n* = 2), the United Kingdom (*n* = 1), France (*n* = 1), and Portugal (*n* = 1). Among them, 100 isolates were derived from human hosts, three from animals, and one had unknown host information. Phylogenetic analysis revealed that the strains most closely related to K31 were two isolates from China (BioSample: SAMN37878107 and SAMN07609103); however, all three strains differed by more than 60 SNPs, indicating a notable degree of genetic divergence. Resistance gene analysis showed that 94.2% (98/104) of the ST145 isolates carried carbapenemase genes, including *bla*_VIM-1_ (*n* = 85), *bla*_KPC-2_ (*n* = 7), *bla*_NDM-1_ (*n* = 7), *bla*_OXA-48_ (*n* = 2), *bla*_KPC-3_ (*n* = 1), *bla*_VIM-4_ (*n* = 1), and *bla*_IMP-96_ (*n* = 1). In addition, the isolates harbored a wide array of other resistance determinants, including genes associated with resistance to rifampicin (*ARR-3*), aminoglycosides (*aac (3)-IIa*, *aac (3)-IId*, *aac(6′)-IIc*, *aadA*, *aph(3′)-Ia*, *aph (6)-Id,* etc), other β-lactams (*bla*_CMY_, *bla*_CTX-M_, *bla*_TEM_, *bla*_DHA_, etc), chloramphenicol (*catA*, *catB*, *cmlA1*), trimethoprim (*dfrA*), macrolides (*mph(A*), *mph(E*), *msr(E*)), quinolones (*qnr*), sulfonamides (*sul1*, *sul2*), tetracyclines (*tet(A*), *tet(B*), *tet(D*)), and colistin (*mcr-9*) ( Fig. 3).

## DISCUSSION

In recent years, there has been a marked increase in the clinical isolation rate of carbapenem-resistant *Enterobacterales* (28, 29). Data from the SENTRY Global Antimicrobial Surveillance Programme (2013–2023) indicate that among 8962 *K*. *oxytoca* isolates, the overall resistance rates to imipenem (1.0%) and meropenem (0.9%) remain relatively low, but the resistance trend is steadily increasing. The imipenem resistance rate increased from 0.4% in 2013 to 1.5% in 2023, while the meropenem resistance rate increased from 0.5% to 1.1% over the same period. In contrast to these global trends, data from the China Antimicrobial Resistance Surveillance System (CARSS) in 2019 indicated significantly higher non-susceptible rates among *K. oxytoca* isolates, with 6.4% and 5.5% non-susceptible to imipenem and meropenem, respectively (2). Carbapenem-resistant *K. oxytoca* was first reported in 2003, with the strain harboring the *bla*_KPC-2_ gene and exhibiting high resistance to both cephalosporins and carbapenems (10). In 2010, a nosocomial infection outbreak in a Spanish intensive care unit was associated with nine *K. oxytoca* isolates carrying *bla*_IMP-8_, which had no or low resistance to carbapenems (30). In the present study, we identified *K. oxytoca* K31, which co-harbors the *bla*_KPC-2_ and *bla*_IMP-96_ resistance genes. This strain demonstrated high resistance not only to imipenem and meropenem, but also to several cephalosporins (ceftazidime, ceftriaxone, cefepime), β-lactam/β-lactamase inhibitor combinations (piperacillin-tazobactam, ceftazidime-avibactam), and quinolones (ciprofloxacin, sitafloxacin). Only amikacin, meropenem-vaborbactam, aztreonam-avibactam, tigecycline, eravacycline, and colistin showed good *in vitro* activity against this strain.

Carbapenemase production is the primary mechanism of carbapenem resistance in carbapenem-resistant *Enterobacterales* (CRE). Zhang et al. (31) studied 1105 non-duplicate CRE strains from China and found that NDM and KPC-2 were the most prevalent carbapenemase types. Among the 24 *K*. *oxytoca* isolates, carbapenemase genes were detected in all strains, including KPC (29%, 7/24), NDM (42%, 10/24), IMP (25%, 6/24), and KPC + NDM (4%, 1/24) (31). There are also significant regional differences in the resistance genes carried by *K. oxytoca* strains. For example, strains in Poland predominantly produce VIM (32), whereas those in the United States are predominantly KPC producers (33). In a multicenter study conducted by Wan et al. in 2023, 25 carbapenem-resistant *K. oxytoca* isolates were collected from five hospitals in Beijing, Chengdu, Guangzhou, Yinchuan, and Suzhou. Carbapenemase genes were detected in 92.0% (23/25) of the isolates, with two strains lacking carbapenemase genes (34). Interestingly, *K. oxytoca* strains carrying *bla*_IMP-8_ isolated in Spain did not show high-level resistance to carbapenems (30), and conjugation experiments in our study further revealed the influence of carbapenemase production on the resistance phenotype. KPC-2-positive transconjugants showed low-level resistance to meropenem and intermediate resistance to imipenem. The *bla*_IMP-96_-positive transconjugants also showed a low level of resistance to meropenem but were sensitive to imipenem. These findings suggest that bacterial resistance is a multifactorial process and that carbapenemase production may be only one component. Alternations in porin structures, overexpression of efflux pumps, and high expression of other non-carbapenem β-lactamases may act synergistically to enhance resistance to carbapenems. In addition, *K. oxytoca* complex strains often carry the chromosomally encoded *bla*_OXY_ gene, which confers low-level resistance to penicillin (35). In our study, strain *K. oxytoca* K31 also carried the chromosomally encoded *bla*_OXY-2-9_ gene and plasmid-borne β-lactam resistance genes, including *bla*_TEM-1B_, the aminoglycoside resistance genes *aac (3)-IId* and *aac(6')-Ib-Hangzhou*, and the quinolone resistance gene *qnrS1*.

Since the first report of the class A serine carbapenemase KPC-1 in *Klebsiella pneumoniae* in 2001 (12), Gram-negative bacteria carrying the *bla*_KPC-1_ gene and its variants have become widespread worldwide, with *bla*_KPC-2_ being the most common subtype. It has been identified in several pathogens, mainly *Enterobacterales* (such as *Klebsiella* spp., *Escherichia coli*, *Citrobacter* spp.) and non-fermenting bacteria (such as *Acinetobacter* spp., *Pseudomonas* spp.) (36, 37). Previous studies have suggested that mobile genetic elements, such as transposons may play an important role in the rapid dissemination of *bla*_KPC_ (38, 39). *bla*_KPC-2_ is often located within the transposon Tn*4401*, which consists of the transposase gene (*tnpA*), the resolvase gene (*tnpR*), and two insertion sequences, IS*Kpn6* and IS*Kpn7* (40). In previously reported *K. oxytoca* isolates, the genetic environment surrounding *bla*_KPC-2_ was characterized as IS*Kpn27-Δbla*_TEM-1_*-bla*_KPC-2_*-Δ*IS*Kpn6*, in which IS*Kpn6* is arranged in parallel with the *bla*_KPC-2_ gene. This structure is presumed to be derived from the Tn*4401* transposon (11). In the current study, the *K. oxytoca* K31 isolate also carried the *bla*_KPC-2_ gene. The surrounding genetic structure of *bla*_KPC-2_ was similar to that of the classical Tn*4401*, but with differences in the insertion sequences. Specifically, *bla*_KPC-2_ was flanked by IS*Kpn27* and an IS*Kpn6-like* sequence. This structure shared 99% homology with the plasmids BSI-KPN166 plasmid p3 and pKP20-558-3, suggesting a possible common evolutionary origin. In addition, downstream of *bla*_KPC-2_, we identified an aminoglycoside resistance gene *aac (3)-IId* flanked by the insertion sequence IS*26*. The genetic structure of this region was very similar to that found in the *Citrobacter braakii* plasmid OT129. The combined presence of *bla*_KPC-2_, *aac (3)-IId*, and surrounding genes formed a novel multidrug resistance gene island. Existing studies have also confirmed that ISs play a crucial role in mediating plasmid sequence rearrangements and horizontal transfer of resistance genes (41, 42). IMP genes were the first metal-type carbapenemase genes to be discovered (43). Members of the IMP gene family are typically integrated into the genetic structure of class one integrons as gene cassettes (44, 45). The *bla*_IMP-96_ gene, which is the focus of this study, is a mutant of IMP-8 that differs by a single nucleotide mutation resulting in a serine-to-glycine substitution at position 214 (S214G). This mutation disrupts the hydrogen bonding with the catalytic residues, thereby enhancing resistance to meropenem. This mutation alters the enzyme’s conformation and disrupts the hydrogen bonding interactions with the catalytic residues, thereby enhancing resistance to meropenem (20). This amino acid substitution has also been observed in *E. coli*, where it confers increased resistance to certain carbapenem antibiotics (46). The *bla*_IMP-96_ gene was first identified on the chromosome of *Stenotrophomonas* spp. isolated in Beijing, China, in 2011. Sequence comparison revealed that similar genetic structures surrounding *bla*_IMP-96_ were also present in *Klebsiella pneumoniae* strain IR5011, which was isolated in the same city and year, and later in plasmid pY-2.p1 of *Enterobacter hormaechei* Y-2 recovered in Hohhot, China. Compared with the original structure in the *Stenotrophomonas* spp. chromosome, the *IntI1* element to the right side of the *bla*_IMP-96_ gene is replaced by a transposase-associated gene cluster. In IR5011, no *IntI1* element was detected upstream of *bla*_IMP-96_, which may be attributed to limitations of sequencing coverage or assembly gaps. Interestingly, although *bla*_IMP-96_ is located at the beginning of the plasmid sequence in pY-2.p1, a complete *IntI1* gene is detected at the other end of the circular structure of the plasmid. Due to the peculiar circular nature of plasmids, *IntI1* is actually located upstream of *bla*_IMP-96_, suggesting that it may play a role in the transfer of the resistance gene. Furthermore, in our study, *Enterobacter hormaechei* strain ECC55, which also carries the *bla*_IMP-96_ gene, showed a genetic environment that was very similar to the genetic structure of IR5011, plasmids pY-2.p1, and pK31-IMP. These findings indicate that this conserved genetic structure may have remained relatively stable during the vertical dissemination of *bla*_IMP-96_ among different bacterial species. Related studies have also reported similar gene structures for plasmids carrying *bla*_IMP-8_, such as *IntI1-bla*_IMP-8_-*aacA4-TniR-TniQ-TniB-TniA* (47). Thus, it remains unclear whether the *IntI1* element carrying *bla*_IMP-96_ originated from a transposition event involving the *IntI1* gene cassette from the chromosome of *Stenotrophomonas* spp. or from a mutation in plasmid-borne *bla*_IMP-8_ sequences during adaptive evolution. However, based on existing research findings, we believe that the *IntI1*-mediated transfer mechanism of *bla*_IMP_ gene cassettes plays an important role in the spread of resistance genes in Gram-negative bacteria. These results suggest that horizontal gene transfer and homologous recombination mediated by mobile genetic elements are key mechanisms for the widespread transfer of carbapenem resistance genes, significantly accelerating the spread of carbapenem-resistant phenotypes in clinical pathogens. In addition, *bla*_IMP_ often coexists with other resistance genes within integrons, which, besides aminoglycoside resistance genes, may also include the class D β-lactamase gene *bla*_OXA_ and the phenicol resistance gene *catB3*(20), leading to multidrug resistance and complicating the selection of effective clinical antimicrobial therapies.

Genomic analysis of global ST145 *K. oxytoca* isolates revealed that 94.2% of strains carried carbapenem resistance genes, and all isolates carried multiple additional classes of antimicrobial resistance genes. These findings indicate that ST145 *K. oxytoca* has emerged as a high-risk, multidrug-resistant clonal lineage. The majority of isolates (79.8%, 83/104) originated from Poland, of which 82 carried *bla*_VIM-1_ and one carried *bla*_VIM-4_. Although only four isolates were reported from Spain, three of them harbored *bla*_VIM-1_, suggesting that *bla*_VIM-1_-positive ST145 strains may be widely disseminated across Europe. Among the 12 isolates from China, the most prevalent carbapenemase gene was *bla*_NDM-1_ (58.3%, 7/12), consistent with findings reported by Zhang et al. (28). Notably, six ST145 isolates were found to co-harbor both class A and class B carbapenemase genes. Among them, four isolates carried both *bla*_VIM-1_ and *bla*_KPC-2_, originating from Poland (*n* = 2) and Spain (*n* = 2). The remaining two isolates were from China, including the K31 strain described in this study and another strain harboring both *bla*_NDM-1_ and *bla*_KPC-2_. In addition, three isolates carried the mobile colistin resistance gene *mcr-9*: one from the United States (harboring *bla*_KPC-3_), one from Spain (harboring *bla*_VIM-1_), and one from Portugal (with no detectable carbapenemase gene). The co-occurrence of carbapenemase and colistin resistance genes in these isolates poses a significant challenge to clinical management.

### Conclusion

In conclusion, our study confirmed the simultaneous presence of the *bla*_KPC-2_ and *bla*_IMP-96_ resistance genes in a *K. oxytoca* strain. These two carbapenemase genes were located on IncFIB(K)-like and IncM1-type plasmids, respectively, both of which possess conjugative transfer capabilities. We also analyzed the genetic structures surrounding the *bla*_KPC-2_ and *bla*_IMP-96_ genes. These results suggest that resistance-encoding genes can be captured by mobile genetic elements, forming stable, heritable composite transposons, integrons, or resistance gene islands, which in turn facilitate horizontal gene transfer between bacterial species. The widespread dissemination of resistance genes carried by mobile genetic elements between bacterial species not only accelerates the evolution of resistant bacteria but also poses a serious challenge to current antimicrobial treatment strategies. In particular, the emergence and potential spread of *Enterobacterales* strains co-producing both *bla*_KPC-2_ and *bla*_IMP-96_ could have serious implications for clinical treatment. We strongly recommend the urgent implementation of effective surveillance systems and stringent infection control measures to prevent potential outbreaks of such pathogens in healthcare settings across China. Based on a global genomic analysis of ST145 *K. oxytoca* isolates, we found that this sequence type exhibits an exceptionally high carriage rate of carbapenem resistance genes and has emerged as a high-risk epidemic clonal lineage with significant public health implications. Enhanced surveillance and screening measures should therefore be implemented in clinical settings to monitor the spread of this lineage.

## Data Availability

The complete genome sequence of K31 was deposited in the National Center for Biotechnology Information database (https://www.ncbi.nlm.nih.gov/) with BioProject number PRJNA1254426 and BioSample number SAMN48111656. The Illumina sequence reads of ECC55 were deposited with BioProject number PRJNA1254870 and BioSample number SAMN48125599.
